# The prevalence and development of digenean parasites within their intermediate snail host, *Galba truncatula*, in a geographic area where the presence of *Calicophoron daubneyi* has recently been confirmed

**DOI:** 10.1016/j.vetpar.2017.03.021

**Published:** 2017-06-15

**Authors:** Rhys Aled Jones, Hefin Wyn Williams, Sarah Dalesman, Sinmidele Ayodeji, Rowan K. Thomas, Peter M. Brophy

**Affiliations:** Institute of Biological, Environmental and Rural Sciences (IBERS), Aberystwyth University, Penglais, Aberystwyth, Ceredigion, SY23 3DA, UK

**Keywords:** *Fasciola hepatica*, Generalized estimation equation (GEE), UK, *Calicophoron daubneyi*, *Haplometra cylindracea*, *Galba truncatula*

## Abstract

During the past decade, rumen fluke (*Calicophoron daubneyi*) has established as a prominent parasite of livestock within numerous European countries. Its development and spread is enabled by the presence of its intermediate snail host *G. truncatula.* However, the dynamics of this stage of the *C. daubneyi* lifecycle is yet to be recorded in numerous northern European countries including the UK. Here, the prevalence of *C. daubneyi* along with *F. hepatica, H. cylindracea* and other parasites infecting *G. truncatula* snails on 10 Welsh farms was recorded using morphological and PCR techniques.

A total of 892 *G. truncatula* snails were collected between May and October 2016. The prevalence of *C. daubneyi* in sampled *G. truncatula* snails (4%) was lower compared to *F. hepatica* (5.6%). No association in prevalence between these species was recorded. *Haplometra cylindracea* was found infecting 8.2% of *G. truncatula* snails, with its prevalence within *G. truncatula* populations negatively associated with *F. hepatica* cercariae prevalence (P = 0.004). Generalized estimation equation (GEE) linear regression models identified the levels of respective fluke eggs shed onto pasture as the main significant association between prevalence levels of both *C. daubneyi* and *F. hepatica* within *G. truncatula* populations (P < 0.001). However, equivalent prevalence levels of *C. daubneyi* and *F. hepatica* within *G. truncatula* populations were associated with higher *C. daubneyi* egg outputs and lower *F. hepatica* egg outputs from livestock grazing the *G. truncatula* habitats. Only one of 36*C. daubneyi* infected *G. truncatula* snails was found harbouring its cercarial stages, a significantly lower proportion compared to the 29 of 50 *F. hepatica* infected *G. truncatula* harbouring its respective cercariae (P < 0.05).

These results signify that *C. daubneyi* may be less adept at infecting and developing in the UK’s native *G. truncatula* populations in comparison with *F. hepatica*. However, *C. daubneyi* has previously demonstrated its ability to progressively adapt to an intermediate host in a new environment. If *C. daubneyi* were to adapt to infect and develop more efficiently in UK *G. truncatula* populations, paramphistomosis risk would significantly increase leading to increased livestock losses. Questions are also raised regarding potential interaction between digenean species at intermediate snail host level, which could impact subsequent livestock trematodosis risk.

## Highlights

• *C. daubneyi, F. hepatica* and *H. cylindracea* all commonly infect UK *G. truncatula*.
• *C. daubneyi* may be less adept at infecting and developing in UK *G. truncatula*.
• Paramphistomosis risk in the UK may increase if *C. daubneyi* can adapt to this host.
• Evidence of interactions between digenean species infecting *G. truncatula*.

## 1 Introduction

In recent years, the UK (Jones et al., 2017) and numerous other European countries (Malrait et al., 2015, Martinez-Ibeas et al., 2016) have witnessed the establishment of rumen fluke infections within their livestock. This is of concern to livestock producers due to rumen fluke’s potential to cause disease and mortality in both cattle and sheep (Mason et al., 2012, Millar et al., 2012).

As a digenean trematode, rumen fluke requires an intermediate snail host to complete its lifecycle. During this stage, the parasites’ miracidia will penetrate and infect a mollusc and use this interior environment to develop, multiply and mature efficiently. The numerous genera and species of rumen fluke within the paramphistome family have differing preferences for intermediate snail host, a factor which mainly determines the potential geographical range of each species. Historically, *Paramphistomum* spp. were regarded as the prominent rumen fluke species in the UK, a genus which uses aquatic planorbid snails as their intermediate hosts (Sey, 1980). However, increasing reports of rumen fluke occurrence during the past decade has coincided with the detection of *Calicophoron daubneyi* as the prominent rumen fluke in UK livestock (Gordon et al., 2013). This finding is significant due to the fact that the predominant intermediate snail host of *C. daubneyi* is *Galba truncatula* (Dinnik, 1962), a snail which is also the predominant host of liver fluke (*Fasciola hepatica*)*. G. truncatula* snails are widespread in the UK, especially on pastures grazed by livestock and thus the potential geographical spread of *C. daubneyi* along with its subsequent degree of contact with livestock in the country is substantial. In Wales, *C. daubneyi* has been shown to be very common in livestock, especially in western areas (Jones et al., 2017) where climate modelling of *F. hepatica* has shown it to be one of the UK’s most prone regions for fasciolosis occurrence due to its climatic suitability for *G. truncatula* (Fox et al., 2011). However, despite PCR confirmation of *C. daubneyi* infecting *G. truncatula* in the UK (Jones et al., 2015), it remains unclear how susceptible the UK’s indigenous *G. truncatula* populations are to *C. daubneyi* infection. Due to the nature of paramphistomosis, where heavy juvenile infections seem to be the prominent cause of disease (Millar et al., 2012), the susceptibility of the intermediate host snail population will be a major factor in determining disease risk due to the necessity for large quantities of metacercariae to be present on pasture to lead to heavy infections.

Potential competition between *C. daubneyi* and *F. hepatica* to infect their common prospective intermediate host may also ultimately influence respective digenean diseases. These parasites have been shown to protect their positions in the snails’ digestive gland, the prime location for development, by predating on subsequent infecting species (Rondelaud et al., 2007). This, along with indirect antagonism, which may be caused by increased snail mortality or reduced nutrient availability associated with dual infections, can fuel population-wide digenean antagonism, where the presence of one digenean parasite within a snail population diminishes the presence of another (Lim and Heyneman, 1972, Combes, 1982). Moreover, *G. truncatula* are also infected by numerous other parasites known to antagonise *F. hepatica*, including trematodes *Echinostoma* spp. (Gordon and Boray, 1970) and *Haplometra cylindracea* (Whitelaw and Fawcett, 1982), and non-trematodes protostrongylid spp. (Hourdin et al., 1993) and oligochaete worm, *Chaetogaster limnaei* (Rajasekariah, 1978). However, detailed information on the potential relationship between all the potential parasites infecting G. truncatula remains scarce, and it is unclear if potential competition or antagonism can affect subsequent infection levels in their respective final hosts.

In this study, the prevalence of *C. daubneyi, F. hepatica* and other parasites within *G. truncatula* populations on Welsh farms were recorded. Data for *C. daubneyi* and *F. hepatica* prevalence was compared with faecal egg counts from livestock grazing on the pasture surrounding collection sites. This was performed with the aim of measuring the establishment and development of *C. daubneyi* within Welsh *G. truncatula* populations, and to record any potential interaction between the various other parasites infecting these snails.

## 2 Materials and methods

### 2.1 Snail collection

Between May and October 2016, a subset of farms (n = 10) identified as being positive for both *C. daubneyi* and *F. hepatica* during a previous study (Jones et al., 2017) were re-visited to study the intermediate host of both parasites. Farms were located in the Welsh counties of Ceredigion (n = 7) and Gwynedd (n = 3), and were visited on four separate occasions during late spring (May – early June), mid-summer (July – early August), September and October. During the first visit, multiple *G. truncatula* habitats in grazed fields were identified. These habitats were searched for adult *G. truncatula* (>4 mm in length) for approximately 20 min during each visit with the snails collected transported to the laboratory for further analyses. Other snail species were found and collected on the study’s farms. However, this paper will only focus on data surrounding the primary intermediate host of *C. daubneyi, G. truncatula.*

Faecal samples from ruminants grazing each *G. truncatula* containing field were also collected. The aim was to gauge the approximate levels of *F. hepatica* and *C. daubneyi* eggs shed onto pasture, and subsequently the level of *C. daubneyi* and *F. hepatica* infective stages that G*. truncatula* snails may be exposed to. Approximately 20 ml of fresh faeces was collected from 20 fresh individual faecal pats from each snail sampled field. When cattle and sheep were grazing in the same field, faeces from both species were collected and kept separate for the following analysis. On return to the laboratory, samples were kept at 4 °C prior to homogenisation and the submission of approximately 20 g of a pooled sample for sedimentation faecal egg count (FEC). For a detailed account of the FEC protocol, see Jones et al. (2017). The numbers of each fluke’s eggs counted during the FEC protocol were recorded as eggs per gram of faeces (EPG).

### 2.2 Detection of snail infection

Within 24 h of collection, each snail was morphologically identified as *G. truncatula* (Macan, 1977) prior to being crushed between microscope slides and viewed under a light microscope. Larval stages of *C. daubneyi*, *F. hepatica* or other digenean species infecting each snail were morphologically identified following Frandsen and Christensen (1984). The tissue of the snail was transferred into a 0.5 ml centrifuge tube, before DNA was extracted using the Chelex^®^ method of Caron et al. (2011) adapted for the inclusion of 3 μl of proteinase K (20 mg/ml, Fisher Scientific, Waltham, USA) prior to initial incubation. Following DNA extraction, the supernatant was diluted 10 times with nuclease free water, with samples negative for fluke by visual inspection pooled in groups of six snails.

A multiplex polymerase chain reaction (PCR) assay was designed and used to confirm the identity of species identified morphologically as *C. daubneyi* or *F. hepatica* and to detect immature infections of each or both flukes in each snail. This PCR was optimised to detect species specific *F. hepatica* and *C. daubneyi* DNA. Primers, F: 5′-GTTTGTGTGGTTTGCCACGG-3′; R: 5′-CTACCCCAAGCAGCCACTAC-3′ (Jones et al., 2017) were used to amplify a region of *C. daubneyi* cytochrome *c* oxidase subunit 1 (*cox*1) gene [GenBank JQ815200], with primers, F: 5′-GCCGGGTCCTCAACATAATA-3′; R: 5′-AGCACAAAATCCTGATCTTACCA-3′ (Martinez-Ibeas et al., 2013) used to amplify a region of *F. hepatica cox1* gene [GenBank AF216697]. Generic mollusc primers based on *G. truncatula* 18S gene [GenBank: Y09019.1] were designed (F: 5′-GGAAAGAGCGCTTTTATTAGTTCAA-3′; R: 5′-CAGAGTCATCGAAGCAACTCCT-3′) using Geneious software (Kearse et al., 2012) to amplify snail DNA as a positive control. For each sample, a 25 μl master mix was created containing 12.5 μl of MyTaq™ red mix (Bioline, London, UK), 80 μM of each parasite primer set, 40 μM of the snail primer set, one μl of the extracted DNA and nuclease-free-water. Each sample was subjected to PCR amplification consisting of an initial denaturation at 95 °C for 2 min followed by 38 cycles consisting of stages of denaturation (30 s at 95 °C) annealing (30 s at 63 °C) and extension (45 s at 72 °C), before a final 10 min extension phase at 72 °C. PCR products were visualised in 1.5% agarose gel stained with GelRed (Biotium, Hayward, USA) along with positive and negative controls. Amplification of a 912 bp 18S band of snail DNA confirmed the DNA isolation and PCR reaction were successful, with 169 bp and 425 bp bands signifying positive species identification for *C. daubneyi* and *F. hepatica,* respectively. In order to test the sensitivity of this newly created PCR assay, 100 ng/μl of DNA extracted directly from both adult *C. daubneyi* and *F. hepatica* was serially diluted ×10 with 100 ng/μl fluke free *G. truncatula* DNA, with each diluted DNA sample submitted for PCR via the newly designed multiplex protocol. The first dilution where the multiplex PCR would not amplify DNA from each parasite would indicate its limit of detection.

A subset of DNA amplicons of the cercariae identified morphologically as *H. cylindracea* (n = 8) were sequenced (ABI 3100, Applied Biosystems, Waltham, USA) to confirm species identity. Primers were designed (F: 5′-ACAGCCGTCAGGCTGCTT-3′, R: 5′-AAATCGGTCCGAAAACAACTG-3′) using Geneious software (Kearse et al., 2012) to amplify a 542 bp region of *H. cylindracea* 28S gene [GenBank: AF151933.1]. The sequenced subset of these *H. cylindracea* amplicons incorporated samples from each farm where the parasite was detected. Sequences were aligned to the amplicon’s reference sequence using Geneious software (Kearse et al., 2012) to confirm species identity.

### 2.3 Statistical analysis

A linear regression model with a generalized estimating equation (GEE) was used to identify factors associated with the prevalence of *C. daubneyi, F. hepatica* and *H. cylindracea* within *G. truncatula* populations of sampled habitats nested within farms. Generalized estimation equation models account for potential correlations within subject and missing data points; both of which were relevant to this data set due to data collection occurring across sequential time points and the variable nature of *G. truncatula* visible presence. The working correlation matrix for data inputted into each GEE model was set as independent, as the lowest Corrected Quasi Likelihood under Independence Model Criterion (QICC) values were associated with models created when this working correlation matrix was specified (Cui, 2007). Candidate models were built using a stepwise backward elimination procedure, where the variables with the highest P values were sequentially removed. Both main effect variables (number of fluke eggs shed onto pasture, number of snails collected, habitat type, pasture type, altitude, farm ID, sampling period and infection prevalence of other fluke species in *G. truncatula* populations) and interaction effect variables (created using two main effect variables) were offered during model creation. The final models were selected via their QICC values (Pan, 2001, Burnham and Anderson, 2002), with the models with the smallest QICC regarded as having the best fit.

To analyse the development of fluke species in *G. truncatula* snails, the proportions of *C. daubneyi* and *F. hepatica* infected snails harbouring the respective cercariae of each species were compared using a Fisher exact test. This analysis was performed on data from each sampling period.

## 3 Results

### 3.1 Prevalence of parasite infection within *G. truncatula*

A total 892 *G. truncatula* snails were collected from 22 habitats within studied farms. Infection prevalence data for these snails are presented in Table 1. Serial dilutions revealed the PCR assay was capable of detecting 0.001 ng of both *C. daubneyi* and *F. hepatica* DNA, a similar amount of DNA as other PCR assays designed to detect fluke infections in *G. truncatula* (Caron et al., 2011, Martinez-Ibeas et al., 2013). However, this PCR assay may have lacked the capability of identifying very low levels of DNA potentially associated with early miracidial infections (Kaplan et al., 1997). The identity of *H. cylindracea* cercariae was confirmed via DNA sequencing, with all amplicons sequenced showing >99% similarity with the 28S reference sequence. Six *G. truncatula* snails were infected with non-digenean parasites, all of which were morphologically identified as *C. limnaei* (Brinkhurst, 1971).

**Table 1:** Prevalence of *F. hepatica, C. daubneyi* and *H. cylindracea* in *G. truncatula* snails and livestock (where applicable) across study period.

	May/Jun	Jul/Aug	Sep	Oct	All
*G. truncatula* collected (n)	217	392	203	80	892
*C. daubneyi* GT prevalence%	2.3	4.1	5.9	3.8	4.0
*C. daubneyi* GT cercariae prevalence%	0.0	0.0	0.5	0.0	0.1
*F. hepatica* GT prevalence%	4.6	5.6	8.4	1.3	5.6
*F. hepatica* GT cercariae prevalence%	1.4	2.8	6.9	1.3	3.3
*H. cylindracea* GT cercariae prevalence%	9.2	7.7	6.9	11.3	8.2
Ruminant *C. daubneyi* mean EPG	20	26	19	23	20
Ruminant *F. hepatica* mean EPG	1	1	7	1	2

GT = *G. truncatula,* EPG = eggs per gram of faeces shed onto pasture.

A total of twenty-two snails (1.88%) were co-infected with two digenean parasites, although none of these were shown to be harbouring cercarial stages of more than one parasite. Two snails were co-infected with *C. daubneyi* and *F. hepatica* (one *F. hepatica* cercariae + *C. daubneyi* DNA, and one *C. daubneyi* *+* *F. hepatica* DNA), with seven and thirteen snails infected with *H. cylindracea* cercariae and *F. hepatica* or *C. daubneyi*, respectively. Infections of two or more digenean parasites within *G. truncatula* populations were seen in 50% of studied habitats. Twenty seven percent of habitats sampled harboured two parasites within the snail population and 23% harboured three parasites. *C. limnaei* was seen co-infecting with *F. hepatica* cercariae in two *G. truncatula* snails.

### 3.2 GEE models

Details on selected GEE models can be seen in Table 2. Final *C. daubneyi* and *F. hepatica* models identified the levels of their respective eggs shed onto snail containing pastures as the only variable significantly associated with the parasites prevalence within *G. truncatula* populations. Both final models had QICC values at least two points lower than the QICC of other candidate models including models where interaction terms were included. Two final *H. cylindracea* models were selected as their calculated QICC values were separated by less than two and thus the superior model could not be identified (Burnham and Anderson, 2002). The first model identified the prevalence of *C. daubneyi* within *G. truncatula* populations as a significant positively associated variable, while the second model identified the prevalence of *F. hepatica* cercariae within *G. truncatula* populations as a significant negatively associated variable.

**Table 2:** GEE linear regression model of factors associated with the prevalence of *C. daubneyi, F. hepatica* and *H. cylindracea* within populations of *G. truncatula* on Welsh farms.

Model	QICC	Variable	β	S.E	95% CI	Wald χ ^2^	Sig.
*C. daubneyi* prevalence%	3.383	Intercept	0.0121	0.0082	−0.004: 0.028	2.17	0.14
		*C. daubneyi* EPG	0.0015	0.0002	0.001: 0.002	42.57	0.000

*F. hepatica* prevalence%	4.538	Intercept	0.0292	0.0131	0.003: 0.055	4.95	0.026
		*F. hepatica* EPG	0.0115	0.0006	0.010: 0.013	418.8	0.000

*H. cylindracea* prevalence% 1	5.607	Intercept	0.068	0.0344	0.000: 0.135	3.89	0.049
		*C. daubneyi* snail prevalence%	0.69	0.1438	0.408: 0.972	23.07	0.000

*H. cylindracea* prevalence% 2	5.802	Intercept	0.109	0.0316	0.047: 0.171	11.97	0.001
		*F. hepatica* cercariae snail prevalence%	−0.297	0.1034	−0.50: −0.095	8.26	0.004

EPG = eggs per gram of faeces shed onto pasture.

### 3.3 *Galba truncatula* infectivity

**Fig. 1:**
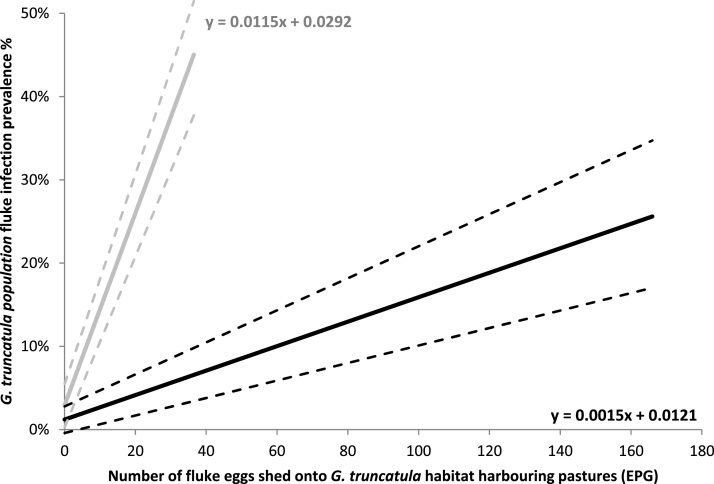
Fitted GEE linear regression equations of *C. daubneyi* (black) and *F. hepatica* (grey) along with their respective 95% confidence intervals (- - -).

Discrepancies between *F. hepatica* and *C. daubneyi* levels in *G. truncatula* snails (where overall *F. hepatica* prevalence was higher) and in grazing livestock (where overall *C. daubneyi* egg counts were higher) were observed and can be seen in Table 1. These discrepancies were highlighted in the regression equations calculated in the final *F. hepatica* and *C. daubneyi* models, and are visualized in Fig. 1.

The proportions of *C. daubneyi* and *F. hepatica* infected snails harbouring their respective cercariae during the sampling periods of July/August and September were significantly higher for *F. hepatica* compared to *C. daubneyi* (Table 3). Higher proportions of *F. hepatica* infected snails harbouring cercariae were also seen in the sampling periods of May/June and October (Table 3), however, these differences were not significant, likely due to the small sample size of infected snails collected during those periods.

**Table 3:** Fisher exact tests for the proportion of *C. daubneyi* and *F. hepatica* infected *G. truncatula* snails harbouring cercarial stages of each respective parasite.

		May/Jun	Jul/Aug	Sep	Oct
*C. daubneyi*	Cercariae harbouring snails	0	0	1	0
	Total infected snails	5	16	12	3
	Percentage of infected snails harbouring cercariae	0	0	8.33	0

*F. hepatica*	Cercariae harbouring snails	3	11	14	1
	Total infected snails	10	22	17	1
	Percentage of infected snails harbouring cercariae	30	50	82.35	100
	**χ ^2^**	1.39	6.877	5.723	1.875
	Sig.	0.520	0.009	0.034	0.400

## 4 Discussion

This study is the first to record *C. daubneyi* prevalence within *G. truncatula* snails in the UK. The recorded prevalence levels of *C. daubneyi* and *F. hepatica* infecting *G. truncatula* snails were within the range of prevalences recorded in studies in France (Abrous et al., 1999, Mage et al., 2002) and Spain (Manga-González et al., 2009, Iglesias-Piñeiro et al., 2016). The GEE models identified the number of respective fluke eggs shed onto pasture as the main association between prevalence levels of both *C. daubneyi* and *F. hepatica* within snail populations. However, equivalent increases in the prevalence levels of *C. daubneyi* and *F. hepatica* within *G. truncatula* populations were associated with larger increases in the numbers of *C. daubneyi* eggs, and smaller increases in numbers of *F. hepatica* eggs shed by livestock onto pastures containing *G. truncatula* habitats. This may indicate that *C. daubneyi* was less effective at infecting sampled snails compared to *F. hepatica, w*hich may be indicative of *C. daubneyi* snail infection prevalence which remained low in comparison to *F. hepatica* despite *C. daubneyi* egg output being on average 10-fold greater than that of *F. hepatica*. This higher burden for *C. daubneyi* in livestock is likely to have occurred due to Welsh farmers’ lack of awareness of, and reluctance to treat rumen fluke infections (Jones et al., 2017); with only one farm in the study indicating that they specifically treat against the parasite. The models also highlight the importance of reducing fluke egg contamination of pasture to reduce the future infective snail population, a practice which is commonly ignored by farmers within their liver fluke treatment regimens (as cited by Claxton, 2015). Yet, it remains to be seen how effective a *C. daubneyi* treatment regimen can be in the UK, with treatment options limited to Oxyclozanide, an anthelmintic that is unlicensed for treatment against rumen fluke in Europe (Malrait et al., 2015) and is known to have variable efficacy against paramphistomes in the absence of multiple administration of the drug (Rolfe and Boray, 1987). Differences in the proportions of *C. daubneyi* and *F. hepatica* infected snails harbouring infective cercarial stages of each respective parasite were also recorded which raise questions regarding the effectiveness of *C. daubneyi* development within sampled snails. Indeed, only one *C. daubneyi* cercariae harbouring *G. truncatula* was identified throughout the study.

Overall, these findings indicate that *C. daubneyi* may be less suited to infecting and developing in the UK’s native *G. truncatula* populations compared to *F. hepatica.* This suggests that at present, the potential for *C. daubneyi* to become a highly problematic parasite in the UK may be limited. As a preferential selfer, *G. truncatula* snails have a high genetic differentiation between populations (Trouvé et al., 2003), a trait which could explain why *G. truncatula* susceptibility to *F. hepatica* can vary significantly between populations, with snails sourced from habitats devoid of natural contact with livestock shown to be more resistant to *F. hepatica* infection and its subsequent development (Rondelaud, 1993). The latter finding may be caused by incomplete adaptation between snail and parasite due to lack of contact (Rondelaud, 1993, Vignoles et al., 2002, Dreyfuss et al., 2012), with digenean/snail adaptation processes reliant on persistent contact (Boray, 1966, Rondelaud et al., 2014, Rondelaud et al., 2015). It remains unclear whether *C. daubneyi* is a new parasite in the UK, or has been present in the country at undetectable levels prior to its recent apparent emergence (Jones et al., 2017). However, even in the latter instance, its historical exposure to the majority of *G. truncatula* populations would have been negligible. With numerous *G. truncatula* populations across the UK now being newly exposed to *C. daubneyi,* it is feasible that the parasite could be in the process of progressively adapting to infect and develop within these snail populations. Ominously, *C. daubneyi* has already demonstrated its ability to progressively adapt to an intermediate host in a new environment. Retrospective analysis of over eighteen thousand *G. truncatula* snails collected in Corrèze, France, revealed prevalence of *C. daubneyi* within these snails had progressively increased between 1989 and 2000 (Mage et al., 2002). Furthermore, free redial burdens within individual infected snails had also increased over the same period, with levels rising to become similar to consistently observed *F. hepatica* redial burdens in the same populations by the final year of the study (Mage et al., 2002). Similar findings have also been observed in two other regions of France regarding *C. daubneyi* prevalence and its infection intensity in *G. truncatula* snails, with the number of *G. truncatula* snails found harbouring three or four sporocyst progressively increasing during a twelve year period from 1994 (Dreyfuss et al., 2008).

If this progressive adaptation was to occur in the UK as well as in other potentially novel geographical areas for *C. daubneyi*, it could have major implications for future paramphistomosis risk. At present, all reports of paramphistomosis in the UK have been attributed to heavy juvenile infections (Mason et al., 2012, Millar et al., 2012, SAC, 2016, Anon, 2016). For this to occur, a ruminant would have to simultaneously ingest large numbers of *C. daubneyi* metacercariae, which in turn would only be present on pastures inhabited by a large population of *C. daubneyi* shedding snails. Currently, high levels of *C. daubneyi* eggs are being shed onto pastures leading to, presumably, a high level of miracidial challenge for *G. truncatula* populations. If *C. daubneyi* was to adapt to the UK’s *G. truncatula* snails, in a similar manner as witnessed in France, conditions may become ideal for paramphistomosis risk to significantly increase. Worryingly, western areas of the UK along with Ireland are regarded as areas within Europe with the best conditions for *G. truncatula* activity, and are inhabited with the highest densities of grazing ruminants (Caminade et al., 2015). Thus, future paramphistomosis losses in these areas could potentially be greater than previously recorded in countries with historic *C. daubneyi* issues.

One possible positive effect of this potential progressive adaptation would be increased competition for *F. hepatica* to infect and develop within *G. truncatula* populations, potentially leading to reduced burdens of its metacercariae on pastures. No significant associations between *C. daubneyi* and *F. hepatica* were recorded during GEE model analysis in this study. However, with *C. daubneyi* still potentially in the process of adapting to its intermediate host in the UK, any possible interactions negating *F. hepatica* may only be prospective. It has been suggested that *C. daubneyi* spread in France was partly fuelled by an increasing efficacy of *F. hepatica* control and the subsequent reduction in *F. hepatica* infected *G. truncatula* snails (Rieu et al., 2007). Moreover, the intensity of *C. daubneyi* infections in cattle herds was shown to be negatively correlated with *F. hepatica* infection intensity in a recent study in Wales (Jones et al., 2017). This latter finding was partly attributed to varying *F. hepatica* treatment efficacy, with *C. daubneyi* hypothesised to have difficulty in establishing and infecting in high intensities on farms where levels of *F. hepatica* were consistently high. This theory would be supported further if *C. daubneyi* is indeed less adept at infecting and developing in the UK’s *G. truncatula* populations compared to *F. hepatica,* especially considering *F. hepatica* is regarded as having dominance over *C. daubneyi* in dually infected snails (Chipev et al., 1985). Co-infections between *C. daubneyi* and *F. hepatica* within *G. truncatula* were recorded, however, these were rare as was seen in comprehensive studies in France (Rondelaud et al., 2004, Rondelaud et al., 2016). This lack of co-infections could be an indication of competition occurring in the field, with secondary infecting fluke species failing to establish in the snail, or its successful infection leading to snail death due to internal pressures (Goumghar et al., 2000). Competition between *C. daubneyi* and *F. hepatica* has already been demonstrated in laboratory studies, with interspecies predation, competition for nutrients and changing biochemical composition of snail tissue all antagonising factors observed when co-infections of both species were initiated (Chipev et al., 1985, Rondelaud et al., 2007).

The most common parasite found infecting *G. truncatula* during the study was *H. cylindracea,* and thus the *F. hepatica* lifecycle may already be affected due to competition on some farms. Information on *H. cylindracea* in the UK is scarce, and thus it is unclear if the relatively high levels observed in these Welsh farms differ from its prevalence both historically and across various regions of the UK. In France, overall prevalence has been shown to be low (Mage et al., 2002, Rondelaud et al., 2016), although levels within individual habitats have been recorded as high as 31.5% (Goumghar et al., 2000). In the latter study, *F. hepatica* prevalence within *G. truncatula* populations was shown to be lower in habitats where *H. cylindracea* was present, while Whitelaw and Fawcett (1982) suggested that high levels of *H. cylindracea* within a Scottish farm’s *G. truncatula* population might have been the cause of the absence of *F. hepatica* in those snails. The negative association recorded in the prevalence of both *H. cylindracea* and *F. hepatica* cercariae within *G. truncatula* populations in this study could be an indication of antagonism. On the contrary, *C. daubneyi* prevalence was positively associated with *H. cylindracea* which potentially indicates a facilitation effect between two parasites, a phenomenon whereby the presence of one infecting parasite may increase a snail’s susceptibility to another infecting species (Combes, 1982). This potential facilitation effect could be an important factor in *C. daubneyi* establishment if indeed UK *G. truncatula* are currently not fully susceptible to *C. daubneyi* infection. However, no snails harbouring cercariae of more than one species were recorded. Laboratory studies have shown that *H. cylindracea* infected *G. truncatula* snails are capable of being infected and fully sustaining a *F. hepatica* or *C. daubneyi* infection through to shedding, however, cercerial output of both subsequent infecting parasites was very low due to indirect antagonistic effects (Moukrim et al., 1993).

*C. limnaei,* a parasite known to predate on (Michelson, 1964, Rajasekariah, 1978) and potentially regulate trematode larvae (Ibrahim, 2007), was also recorded infecting *G. truncatula* snails. Considering its rarity and its presence co-infecting *G. truncatula* with *F. hepatica* cercariae, it is unlikely that *C. limnaei* had any major negating effects on *F. hepatica* prevalence within sampled snails, although this could differ if *G. truncatula* populations commonly infected with *C. limnaei* exist. Indeed, it remains to be seen whether the presence of any parasite including *H. cylindracea*, or a fully adapted *C. daubneyi* can have any major negating effects on subsequent livestock *F. hepatica* infections, with no known research on this aspect found in the literature. Spatial modelling of *F. hepatica* at herd level has been shown to be less accurate compared to regional models, with numerous unknown localised variations believed to cause a prevalence disparity in small regions exposed to the same climate variables (Howell et al., 2015). If *H. cylindracea* does indeed have an antagonistic effect on *F. hepatica,* it could be one of many factors causing these localised variations, with *H. cylindracea* prevalence shown to be capable of varying significantly within *G. truncatula* habitats in neighbouring fields in this study. However, populations of the definitive hosts of *H. cylindracea*, toads (*Bufo bufo*) and frogs (*Rana temporaria*), have both been shown to be in decline in the UK (Teacher et al., 2010, Petrovan and Schmidt, 2016), which subsequently may cause a reduction in *H. cylindracea* prevalence within *G. truncatula* populations. This, on some farms at least, may indirectly heighten *F. hepatica* prevalence within its *G. truncatula* populations and subsequent fasciolosis risk.

## 5 Conclusion

This study demonstrates that *C. daubneyi* is infecting *G. truncatula* snails in the UK, although questions remain regarding its capabilities to infect and develop within these populations efficiently. However, evidence suggests that *C. daubneyi* could adapt to become more suitable to this intermediate host. If so, the risk of paramphistomosis could increase significantly in the future, and thus further research on *C. daubneyi* epidemiology is imperative in the eventuality of this scenario. The study also recorded high levels of *F. hepatica* and *H. cylindracea* infecting *G. truncatula* snails on Welsh farms. This raises numerous questions regarding potential interactions at intermediate host level between the three digenean parasites studied and potentially other parasite groups as well. These interactions may involve an antagonistic effect which in theory could be a novel method of negating livestock losses due to trematodosis.
